# Combining the Effects of Mobilization With Movement and Cyriax Physiotherapy in Lateral Epicondylitis: A Case Report

**DOI:** 10.7759/cureus.87093

**Published:** 2025-07-01

**Authors:** Sivakumar S, Ravindra Reddy C, Prakash Jayabalan

**Affiliations:** 1 Department of Physiotherapy, KMCH College of Physiotherapy, Kovai Medical Centre Research and Educational Trust, Affiliated to The Tamil Nadu Dr. M.G.R. Medical University, Coimbatore, IND

**Keywords:** chronic pain, cyriax physiotherapy, lateral epicondylitis, manual therapy, mulligan’s mobilization

## Abstract

Tennis elbow, or lateral epicondylitis (LE), is characterized by a gradual onset of pain over the lateral aspect of the elbow, particularly during wrist extension with a pronated or supinated hand. It is often exacerbated by gripping activities. Common symptoms include pain at the origin of wrist extensor tendons, reduced grip strength, and impaired upper limb function. The etiology may include trauma, degeneration, repetitive grasping, scar tissue formation, or contractures. Diagnosis is based on patient history, functional tests, and palpation of the lateral epicondyle. A 48-year-old woman, a homemaker complained of right elbow pain and swelling and was diagnosed with LE. Pain intensity was assessed with a Visual Analogue Scale (VAS) and functional ability with Patient-Rated Tennis Elbow Evaluation (PRTEE). This case report aims to evaluate the combined effect of mobilization with movement (MWM) and Cyriax physiotherapy on pain reduction and functional improvement in a patient with LE over a two-week intervention period. As a result of physical therapy sessions, pain levels decreased substantially, from 8/10 to 2/10 on the VAS, and improvement in functional ability went from 81/100 to 34/100 in PRTEE. This case suggests that combining MWM and Cyriax physiotherapy techniques may enhance pain relief and functional improvements in patients with LE. Further research with larger statistically significant samples is needed to confirm these findings.

## Introduction

Lateral epicondylitis (LE), commonly referred to as tennis elbow, is a prevalent overuse injury that causes pain and tenderness over the lateral epicondyle of the humerus. It frequently affects individuals whose occupations involve repetitive forearm motions. While the precise etiology remains unclear, it is often associated with microtrauma or overuse, leading to decreased vascularity and delayed healing in the affected area. Histologically, LE is marked by disorganized collagen, increased fibroblast proliferation, and vascular hyperplasia at the extensor tendon origin. Symptoms may persist for months or even years in some cases. LE is characterized by macroscopic and microscopic tears in the extensor carpi radialis brevis tendon, typically resulting from repetitive wrist extension and eccentric muscle contractions (muscle lengthening under tension). The condition affects approximately 1% to 3% of the general population, with peak incidence between 45 and 54 years of age. Both genders are equally affected, though individuals using vibrating tools or engaged in high-demand occupations are at greater risk. Pain typically localizes at the lateral epicondyle but may radiate to the shoulder or wrist in severe cases [1,2].

There are physical interventions available, including electrotherapy like extracorporeal shock wave therapy, ultrasound, and exercise, but the efficacy of these is reported to be low, and larger trials are needed [3]. However, trials have shown greater benefits for manipulation and exercises compared to corticosteroid injections [4]. Cyriax physiotherapy, a combination of deep transverse friction massage and Mill's manipulation, has been shown to alleviate symptoms when applied in a specific sequence [5,6]. Similarly, Mulligan’s Mobilization with Movement (MWM) involves a sustained lateral glide of the elbow during functional movement, effectively reducing pain and improving joint mobility [7,8]. While a standalone manipulation over four weeks typically yields favorable outcomes, we aimed to investigate whether a combined manipulation approach offers additional benefit in pain recovery and functional improvement in patients with LE over a two-week period.

## Case presentation

A 48-year-old woman, a homemaker, presented with persistent right elbow pain and swelling for five months. Initial self-management with analgesics and ice provided only temporary relief. Recurrences were frequent, especially after lifting heavy objects or performing gripping tasks, significantly affecting her daily tasks, such as house cleaning and washing utensils. Upon clinical evaluation by a medical doctor, the patient presented with characteristic signs and symptoms and physical examination findings like tenderness and positive provocative tests consistent with LE. Management initially included the prescription of corticosteroids and the use of a tennis elbow strap, which the patient utilized intermittently. However, the pain persisted, and she was referred to physiotherapy.

At the time of evaluation, the patient reported pain aggravated by gripping and lifting, accompanied by localized swelling and tenderness below the LE. Palpable trigger points and edema were noted. The diagnosis was confirmed based on physical tests, Mill's and Cozen’s test showing positive for tenderness and pain. Functionally, she had difficulty with household chores involving wrist movements. Range of motion was not significantly affected, but it was painful, as reported in Table 1. The Patient-Rated Tennis Elbow Evaluation (PRTEE) scale is a 15-item questionnaire to measure pain (0-50) and functional disability (0-50), with total scores ranging from 0-100, indicating no pain/disability to maximum pain/disability [9]. The PRTEE total score was 81/100, confirming significant disability, and pain intensity was rated 8/10 during activity and 2/10 at rest on the Visual Analogue Scale (VAS) (Table 2). These findings supported the initiation of physiotherapeutic management. Informed consent was taken from the patient for further management.

**Table 1:** Pre- and post treatment range of motion

Joints (active)	Right (pre-treatment)	Right (post treatment)
Elbow flexion	0-125°	0-130°
Elbow extension	0°	0°
Elbow pronation	0-75°	0-80°
Elbow supination	0-60°	0-80°
Wrist flexion	0-72°	0-75°
Wrist extension	0-°60°	0-72°
Radial deviation	0-21°	0-25°
Ulnar deviation	0-33°	0-35°

**Table 2:** Outcome measures pre- and post treatment Visual Analogue Scale (VAS) and Patient-Rated Tennis Elbow Evaluation (PRTEE)

Outcome metrics	Pre-intervention	Post intervention
VAS at movement	8/10	2/10
VAS at rest	2/10	0/10
PRTEE	81/100	34/100

Physiotherapy management

**Figure 1:**
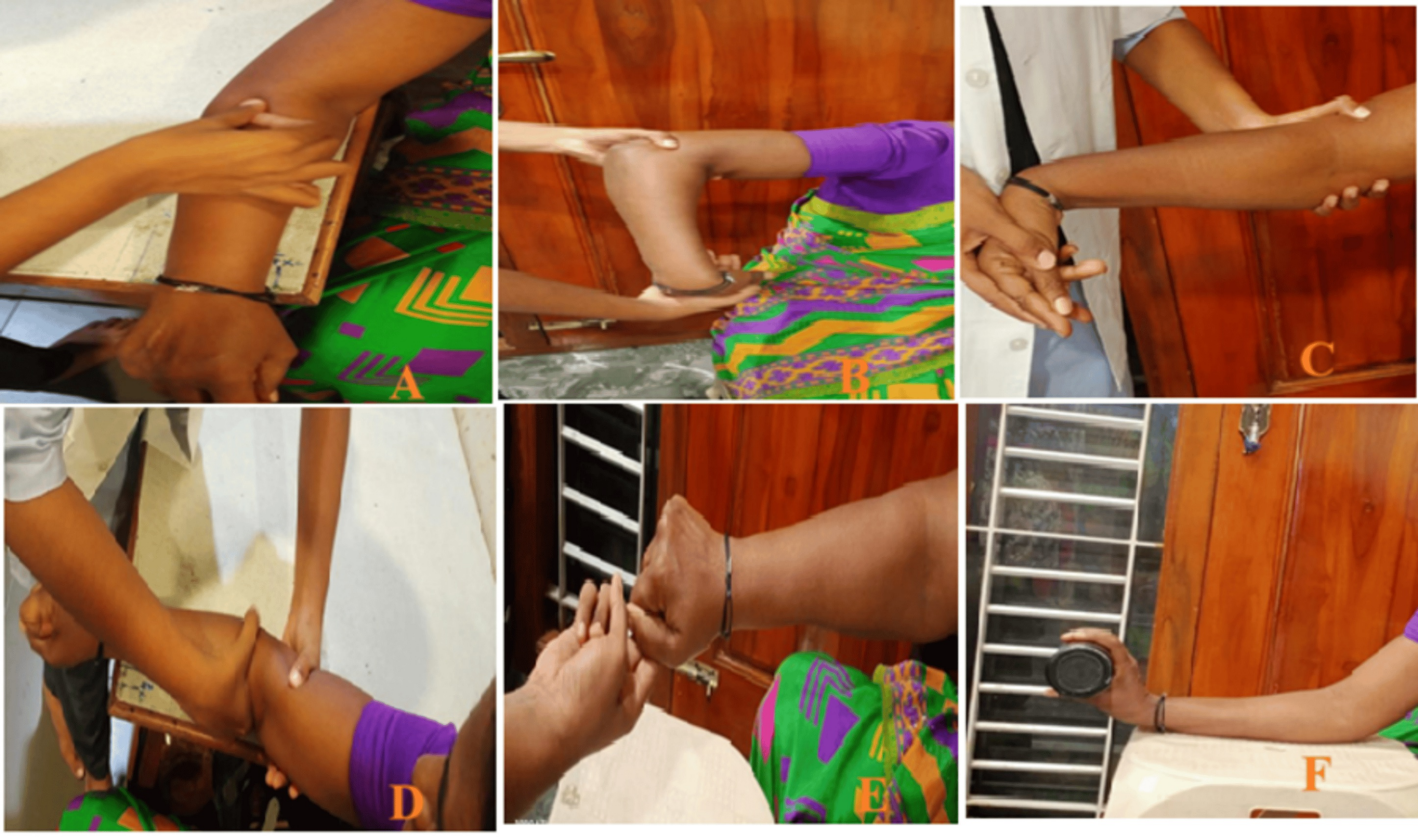
Physiotherapy interventions (A) Cyriax technique; (B, C) Mill's manipulation; (D) Mobilization with movement (MWM); (E, F) Isometric and eccentric strengthening exercises

A comprehensive physiotherapy management plan was developed, comprising Cyriax therapy (deep friction massage) (Figure 1A), followed by Mill's manipulation (Figures 1B, 1C) and MWM (Figure 1D), and strengthening exercises like isometric (Figure 1E) and eccentric (Figure 1F) were given with an adequate rest period (15-30 minutes) between treatments. Treatment duration, sessions, and days are presented in Table 3.

**Table 3:** Physiotherapeutic intervention and protocol for two weeks

Goals	Intervention	Procedure	Rationale	Dosage
To reduce pain and to correct dysfunction	Movement With Mobilization (MWM)	Manually glide the elbow laterally while the subject grips the object.	To reduce pain and improve range of motion (ROM)	Five repetitions, two sets per day, three days per week for two weeks
	Cyriax physiotherapy with Mill's manipulation	The tendons were given deep friction before being manipulated with limited amplitude and high velocity thrust	Elongates the scar tissue and makes the area mobile and pain-free	10 to 15 minutes of friction massage followed by one manipulation and two sets per day, three days per week for two weeks
To improve muscle strength and enhance elbow stability	Isometric strengthening	Instruct the patient to contract the wrist extensors without moving the joint for five to ten seconds and relax	Strengthens without exacerbating pain, maintains muscle function	Two times per day, three sets of 10 to 15 repetitions, two days per week
	Eccentric strengthening with a 5 kg weight	Wrist in flexed position holding a 5 kg weight, slowly raise the wrist into extension. Return to the starting position	Improves strength and flexibility in the wrist, forearm, and elbow	Two times per day, three sets of 10 to 15 repetitions, two days per week

## Discussion

LE affects approximately 1% to 3% of the general population, with a slightly higher prevalence among women [10]. It presents as lateral elbow pain, grip weakness, and functional impairment, impacting daily tasks. Diagnosis is typically confirmed through clinical tests that elicit tenderness over the lateral epicondyle, including resisted wrist extension and passive wrist flexion [11]. Various non-invasive therapeutic interventions are available to treat pain or tenderness for LE. Evidence shows that manual therapy techniques and comprehensive strengthening exercise programs yielded better improvements [12]. In our case, we evaluated the effectiveness of the combined two manual therapy techniques. The patient underwent a two-week intervention combining Cyriax's friction massage, Mulligan’s lateral glides, and strengthening exercises (usual). Cyriax therapy, through deep friction and Mill's manipulation, aims to disrupt adhesions and enhance mobility by lengthening scar tissue. MWM, as proposed by Mulligan, involves an accessory glide during active movement, helping reduce pain and improve function. Post-treatment outcomes showed notable improvements, with a 75% reduction in VAS pain score and a 58% improvement in PRTEE functional scores, with no adverse events reported.

When used separately, both Cyriax and MWM typically require a four-week protocol [6,13,14,15] for better outcomes. Our study results suggest that integrating Cyriax, MWM, and isometric exercises may provide a faster and enhanced recovery compared to using either technique in isolation [16] and also support the recommendations of incorporating multimodal interventions in managing lateral epicondylitis [12]. Notably, the combined approach yielded substantial results within two weeks, indicating potential for faster rehabilitation. However, although the combined technique offers temporary pain relief, there remains a possibility of future exacerbation. The patient was discontinued for long-term follow-up after she had a transient recovery. This study is limited by its design, and future trials with a larger sample size and robust randomized controlled trials are warranted to find combined efficacy.

## Conclusions

The observed reduction in VAS and PRTEE scores indicates that this combinational manual therapy approach can accelerate recovery within a short treatment duration. While the results are promising, they should be interpreted cautiously since it is a single case study. Future trials with larger sample sizes and longer follow-ups are needed to validate the efficacy and sustainability of these combined manual therapy techniques in managing LE.
